# Clinical reliability of radial forearm free flap in repair of buccal defects

**DOI:** 10.1186/1477-7819-11-26

**Published:** 2013-01-30

**Authors:** Qi-Gen Fang, Zhen-Ning Li, Xu Zhang, Fa-Yu Liu, Zhong-Fei Xu, Chang-Fu Sun

**Affiliations:** 1 Department of Oromaxillofacial-Head and Neck Surgery, School of Stomatology, China Medical University, No. 117, Nanjing North Street, Heping District, 110002, Shenyang, Liaoning, People’s Republic of China; 2 Department of Oral and Maxillofacial Surgery, School of Stomatology, China Medical University, No. 117, Nanjing North Street, Heping District, 110002, Shenyang, Liaoning, People’s Republic of China

## Abstract

**Background:**

The ideal method for buccal defects should provide good outcome of both function and appearance; our goal is to highlight the reliability of radial forearm flap in buccal reconstruction.

**Methods:**

A retrospective study was conducted. From 2005 to 2012, 20 radial forearm flaps were used to repair the defects. We analyzed the superiority and reliability of the flap; in addition, we reviewed some related literature and made a comparison between radial forearm flap and platysma flap.

**Results:**

All radial forearm flaps totally survived, but two flaps suffered venous obstruction, hematoma, respectively. Radial forearm flap preserved the original interincisal distance well. In our follow-up, all patients had sufficient mouth-opening width (mean: 4.3 cm).

**Conclusion:**

Radial forearm flap is a reliable method for buccal defect reconstruction.

## Background

Buccal squamous cell carcinomas are the most common malignant tumors among all buccal neoplasms. These carcinomas pose significant threat to patients’ lives and severely affect their quality of life [1]. Traditionally, the treatment strategy for buccal carcinoma is mainly surgery-based comprehensive therapy [2]. Extensive and complete resection of buccal mucosa tumor is a current and reliable method to improve local control of the rate of buccal mucosa carcinoma [3]. The objectives in the reconstruction of buccal defects after surgical resection include restoration of function and structural cosmesis [4]. The radial forearm free flap (RFFF) was first introduced by Yang *et al*. in 1981 [5]. Nowadays RFFF is a workhorse in reconstructive head and neck surgery. It has some well-known advantages: a reliable anatomy, long pedicle length, good-size vessels, suitable thinness and relative sparsity of hair, to substitute mobile oral mucosa [6-8]. In this study, we review our experiences with use of RFFF for buccal defects in a series of 20 patients. The structural and functional advantages of RFFF, including its usefulness and versatility, are presented and discussed.

## Methods

The institutional research committee had approved our study.

Between 2005 and 2012, 20 patients in the department of Oromaxillofacial-Head and Neck Surgery, China Medical University, were treated with RFFF for buccal defects. Medical records were systematically reviewed for all 20 patients. The only exclusion criterion was inadequate information. The defects varied from the retromolar trigone to the oral commissure. In the group that underwent RFFF, the mean age was 58.0 (range 25 to 78) years, and the male–female ratio was 9:11. Seven patients were staged as T2, three as T3, and nine as T4; four patients received postoperative radiation, Through-and-through resections were conducted in three patients, and eleven patients underwent resection of either the mandible or maxilla (Table 1).

**Table 1:** Patients’ summary

**Patient number**	**Age, years**	**Sex**	**Cause**	**Tumor stage**	**Flap size, cm**^**2**^	**Complications**
1	78	M	Precancerous lesion		5.0 × 8.0	No
2	55	F	SCC	T3	5.0 × 7.0	Venous obstruction
3	52	F	SCC	T2	6.0 × 10.0	No
4	61	F	SCC	T4	4.0 × 8.0	No
5	58	M	SCC	T4	9.0 × 15.0	No
6	59	F	SCC	T4	6.0 × 7.5	No
7	59	F	SCC	T2	6.0 × 6.0	No
8	56	M	CCC	T4	6.0 × 6.5	No
9	67	F	SCC	T4	6.0 × 6.5	No
10	44	F	SCC	T3	6.0 × 7.0	No
11	63	M	SCC	T4	7.0 × 7.0	No
12	62	F	SCC	T4	6.5 × 12.0	Hematoma
13	60	M	SCC	T2	6.5 × 11.0	No
14	50	F	ACC	T2	7.0 × 12.0	No
15	25	M	SCC	T2	6.0 × 10.0	No
16	67	F	SCC	T4	6.0 × 6.5	No
17	56	M	SCC	T4	5.5 × 7.0	No
18	77	M	SCC	T2	5.0 × 7.0	No
19	59	F	SCC	T3	6.5 × 10.0	No
20	52	M	SCC	T2	6.0 × 8.0	No

M, male; F, female; SCC, squamous cell carcinoma; CCC, clear cell carcinoma; ACC, adenoid cystic carcinoma.

In all cases, simultaneous flap elevation and recipient-site surgery were performed to shorten the total operation time. Closure of the donor site was performed by a split-thickness skin graft from the upper leg or a full-thickness skin graft from the abdomen. The patients further had a lower-arm splint for immobilization, and the first bandage change was performed on the fifth day postoperatively. Postoperative flap control was performed hourly during the first 24 hours and then every 4 hours for the next 2 days.

In order to show the reliability of RFFF more clearly, we compared the RFF group with the platysma flap (PF) group, in which all the patients were diagnosed with buccal carcinoma and received a primary PF reconstruction. The choice of an RFFF or a PF was based on the patient’s status, the defect size and the experience of the surgeon. In the PF group, there were 24 patients, the mean age was 72.4 (range 55 to 80) years, and the male–female ratio was 11:13. Three patients were staged as T1, twelve as T2, six as T3, and three as T4. Five patients received postoperative radiation, and seven underwent resection of either the mandible or maxilla, and the flaps developed partial necrosis in two patients.

All patients in both groups received open-mouth width measurements. The open-mouth width was defined as the midline distance between the upper margin of the lower gum and the lower margin of the upper gum, and was measured preoperatively and at least 6 months postoperatively.

The Student’s *t*-test was used to study the statistical difference of the variables. A *Ρ*-value less than 0.05 was considered significant.

## Results

All RFFFs survived completely. The mean flap size was 53.6 (range 32 to 135) cm^2^, one flap suffered a venous obstruction, but this situation gradually returned to normal without any surgical treatment; a hematoma developed in another flap but and the flap was salvaged on immediate re-exploration (Table 1).

There was no significant difference between the two groups in preoperative open-mouth width (*P* = 0.73), however, the postoperative distance was wider in the RFFF group than in the PF group (*P* = 0.002), and the change in open-mouth width was significantly smaller in the RFF compared to the PF group (*P* < 0.001) (Table 2).

**Table 2:** Open-mouth width comparison

	**Preoperative distance, mean, cm**	**Postoperative distance, mean, cm**	**Change, %**	**Mean ± SD, %**
RFFF	1.5-6.2 (4.6)	1.4-5.8 (4.3)	4.0-9.1	6.7 ± 1.6
PF	1.2-6.2 (4.8)	1.1-4.7 (3.2)	8.3-47.5	30.4 ± 9.7

RFFF, radial forearm free flap; PF, platysma myocutaneous flap.

## Discussion

There are many methods suitable for repairing defects of the buccal mucosa [1]. Sufficient open-mouth width must be obtained for satisfactory recovery, because it plays a key role in swallowing and articulation. Some local and regional flaps are suggested. The submental artery island flap was first introduced by Martin *et al*. in 1993 [9], Many authors have demonstrated its advantages: excellent skin match, ease of raising the flap and so on, however, this flap has some limitations for intraoral reconstruction: the thickness of the flap and the hair-bearing nature of the region in male patients; marginal mandibular nerve paresis, and potency for increasing regional lymph node metastasis [10-13]. Pectoralis major myocutaneous flaps are well-developed flaps and usually used for defects in the head and neck; however, they are not suitable for minor tissue defects, and are sometimes too corpulent to allow precise moulding in the reconstruction. In addition, these flaps are also not recommended for use in adolescent female patients because of their significant breast-deforming effects [10].

The PF flap has been proven especially suitable for buccal defects. It has many advantages [14-17]: first, preparation of a PF is simple; second, it has a wide arc of rotation and can reach the recipient site easily; third, it can be conducted without microvascular anastomosis. In addition, it has low requirements in terms of the patient’s status, hence it may be more available for older people; in this study, we found that patients in the PF group were significant older than in the RFFF group (Chi square (*χ*^2^) test, *P* < 0.001). However, the PF also has apparent shortcomings: first, blood supply and venous drainage of a PF are uncertain, which may lead to partial or total necrosis (in our study, flaps developed partial necrosis in two patients); second, submandibular lymph node metastasis may preclude the use of PF, and if positive lymph nodes are suspected before, or are identified during surgery, a PF is not recommended; third, PF may be suitable only for small-to-middle sized defects - in our study we found the distribution of tumor stage was significantly different between the two groups (*χ*^2^ test, *P* = 0.046), with most tumors in the PF group staged as T2. In addition, in our study, buccal reconstruction with the PF produced unpredictable results in preserving the original open-mouth width.

The ideal method for buccal defects should provide stable and continuous coverage, acceptable function and cosmetic, minimum morbidity of the recipient and donor site in terms of color, thickness and texture. Advances in microsurgical techniques make this possible. Since Yang *et al*. [5] first described RFFF in 1981, this flap has become increasingly popular in oral and maxillofacial surgery for soft tissue defects. Despite its common use for tissue defects in clinical series, few reports have focused on elaborating the reliability of RFFF in buccal mucosa defects. This flap has many superiorties: we can easily acquire a pedicle longer than 10 cm, which is enough for buccal defects without any limitation; the mobility, pliability and thinness of the RFFF make it the ideal method for buccal reconstruction; the procedure of elevating the flap is easy and the vessel diameter is suitable for anastomosis with a high success rate. The RFFF can also be folded [18]; in our study, we used three folded free radial forearm flaps to repair full-thickness defects, and achieved satisfactory open-mouth width (4.2 cm, 4.6 cm and 4.3 cm).

In our study, venous obstacle occurred in one patient, but this situation gradually returned to normal without any surgical treatment. A hematoma was found in another patient within 24 hours after surgery; we immediately conducted a re-exploration and confirmed it was caused by an anastomosis insufficiency, so we performed a revascularization.anastomosis and the flap was salvaged. Thus, the flap survival rate was 100%. Kruse *et al*. [6] reported that the success rate of RFFF was more than 95%, Shibahara *et al*. [19] reported a total survival rate for RFFF of 100%, and Song *et al*. [1] reported a total survival rate for RFFF greater than 90%. It emphasizes the high reliability of RFFF for treatment of buccal defects. In addition, in our follow-up, most patients had sufficient open-mouth width (mean 4.3 cm), and change in width in the RFFF group was small and significantly smaller than that in the PF group. In a controlled study. by Chien *et al*. [20], the authors suggest that reconstruction with RFFF for buccal mucosal defects is more likely to preserve the original open-mouth width, so we may conclude that the RFFF can protect open-mouth width very well. In addition, we must preserve the mastication muscles purposively during surgery, and postoperative mouth-opening exercises are also very important.

## Conclusion

Compared to the PF, the RFFF offers a series of advantages. RFFF has a high success rate, can achieve sufficiently wide mouth-opening postoperatively, can preserve the original open-mouth width, and is a reliable method for treatment of buccal defects.

## Abbreviations

PF: Platysma flap;RFFF: Radial forearm free flap

## Competing interests

The authors declare that they have no competing interests.

## Authors’ contributions

Q-GF has contributed to the conception, design, and acquisition of information and has written this paper. Z-FX, Z-NL and XZ have contributed to analysis and interpretation of data. F-YL and C-FS have helped draft the manuscript and revise the paper. All authors read and approved the final manuscript.
